# Who controls the tariffs of a human cell?

**DOI:** 10.1038/s44320-025-00112-6

**Published:** 2025-05-12

**Authors:** Maximilian Billmann

**Affiliations:** Institute of Human Genetics, University of Bonn, School of Medicine and University Hospital Bonn, Bonn, 53127 Germany

**Keywords:** Membranes & Trafficking

## Abstract

Four studies in this Mol Syst Biol issue map the first virtual landscape of SLC biology, presenting data from metabolomics/transcriptomics (Wiedmer et al, 2025), proteomics (Frommelt et al, 2025), pertomics (Wolf et al, 2025), and their integration (Goldmann et al, 2025).

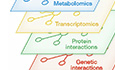

## Teaser

The cell, the functional unit of life, creates well-controlled biochemical environments through partition via membranes and transport of molecules across those membranes via transporters. The human genome encodes for 1500–2000 of such transporters with the largest family, the solute carriers (SLC), comprising around 450 proteins. Despite their crucial physiological role, the large number of disease-linked SLCs, as well as their role as prominent drug targets, as much as one-third of all SLCs lack functional annotation. And even for SLCs with functional annotation, how exactly they act in their complex network to precisely maintain intracellular environments is only anecdotally understood (César-Razquin et al, 2015). To tackle this shortcoming and exploit the immense therapeutic potential, the Resolute consortium built a first virtual landscape of SLC biology by recording and integrating several layers of SLC-centered omics networks. In this issue of *Molecular Systems Biology*, four studies present the central data generation efforts covering metabolomics and transcriptomics (Wiedmer et al, 2025), proteomics (Frommelt et al, 2025), perturbation omics (pertomics) (Wolf et al, 2025), as well as an integration of these data (Goldmann et al, 2025).

To connect SLCs to their substrates, the metabolites they transport across membranes, Wiedmer and colleagues generated experimental cell systems to control the expression of each of 378 SLCs. About half of those they studied through knockout and overexpression, and the remaining SLCs with low endogenous expression levels through overexpression (Wiedmer et al, 2025). Upon overexpression, the authors performed targeted quantification of 189 metabolites representing the metabolome with a small blind spot in lipid metabolism. This impressive effort connected 71 previous orphan SLCs without a clear known substrate to putative substrates or metabolic pathways.

Taking a complementary perspective at metabolic pathways putatively connected to SLCs, Wiedmer and colleagues also recorded differential mRNA expression upon overexpression of each of 441 SLCs showing that the vast majority of metabolic pathways respond to SLC expression levels. To further explore the network of mRNA products, the proteins that are connected to SLCs and thereby putatively modulate and support their function, Frommelt and colleagues generated a tour-de-force interaction proteomics map (Frommelt et al, 2025). They tagged 396 SLCs in HEK293 cells and subjected them to a refined affinity-purified mass spectrometry (AP-MS) protocol, which allows for sensitive detection of physical protein-protein interactions (PPI) at the cytoplasmic and organelle membranes. Overall, this identified about 19,000 PPIs involving 4000 proteins, 96% of those PPIs novel. While PPI profiles were sparser than metabolomics or transcriptomics profiles, they allowed for clustering of SLCs as well and provided another unique omics layer for the virtual embedding of SLCs.

The network of proteins supporting SLC function may contain components lacking direct physical contact with SLCs. Such functional connections can be systematically mapped by measuring genetic interactions (GI), where the combinatorial perturbation phenotype of two genes, for instance two SLCs, cannot be inferred from perturbing each gene alone, because they act in the same or compensatory pathways. Wolf and colleagues comprehensively mapped GIs between SLCs via combinatorial CRISPR-based knockout screening (Wolf et al, 2025). In HCT116 cells, the cell system used by Wiedmer and colleagues to map metabolic and transcriptome signatures for SLCs, Wolf and colleagues targeted all possible pairs between the 258 expressed SLCs, as well as around 2000 SLC-Enzyme pairs, totaling 35,421 tested gene pairs among which they identify 1236 GIs. While this provides a useful resource, particularly in combination with other SLC omics data generated in the Resolute consortium, a few questions beg to be explored. For instance, in line with earlier studies (Shi et al, 2022) Wolf and colleagues demonstrate that GIs between SLCs and metabolic enzymes vary substantially in altered experimental conditions, including hypoxia or drug-mediated inhibition of the respiratory chain. This suggests that similar experimental conditions may provide important insights when measuring SLC-SLC GIs, or, since a fraction of GIs likely also reflects physical interactions, AP-MS-based PPI mapping as presented by Frommelt and colleagues. And just like linking SLCs to physical interaction partners or expression changes (Wiedmer et al, 2025; Frommelt et al, 2025), GI maps between SLCs and the entire genome would be an ambitious next challenge to map the network of proteins supporting SLC function.

The metabolomics, transcriptomics, proteomics, and pertomics reported in this issue are layers of a first comprehensive systematic representation of SLC biology. Goldmann and colleagues integrated those layers with orthogonal information and presented the data in an intuitively browsable, open-access fashion (Goldmann et al, 2025). Goldmann and colleagues included data from previous work by the Resolute consortium covering tertiary SLC structure and genetic variants of SLCs linked to disease phenotypes (Ferrada et al, 2024), they extended SLC previous substrate annotations (Meixner et al, 2020), and defined subcellular SLC localization using their tagged constructs in HEK293 cells (Wiedmer et al, 2025). By adding tissue expression information from the Human Protein Atlas, they showed that almost two-thirds of all SLCs are expressed in a given tissue, with high-energy-demanding tissues such as the brain expressing higher levels of SLCs (Uhlén et al, 2015). Despite the fact that GI networks in the model system yeast map functional similarity between genes very well (Forster et al, 2022), Goldmann and colleagues did not utilize the SLC GI data to inform the functional landscape of human SLCs.

**Figure 1:**
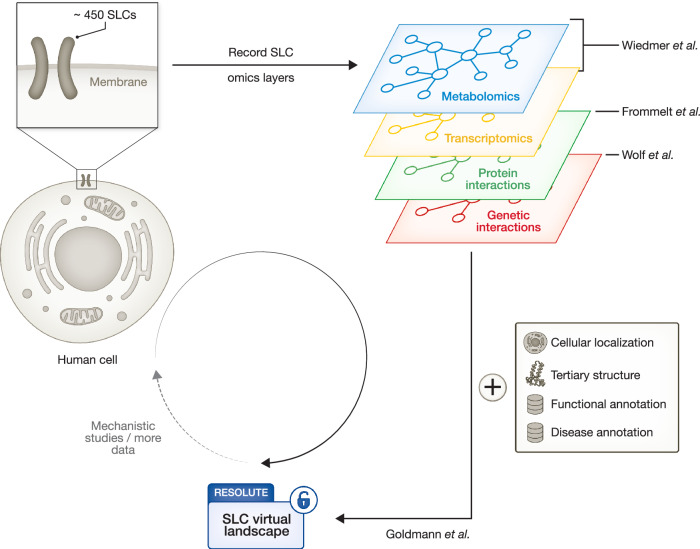
Recording omics layers of solute carriers (SLC). For the approximate 450 SLCs in the human genome, different sub-projects of the Resolute consortium have generated genetic tools and recorded targeted metabolomics, transcriptomics, protein interactions, and genetic interactions. This omics data was integrated with additional information on SLC function and disease association to generate a reference virtual landscape for SLC biology.

Going forward, this open-access multi-omics data resource, along with the generated experimental models, will provide an invaluable discovery tool for in-depth cell biological and biochemical exploration of SLC biology. This data may also be the basis for understanding SLC biology in what is termed a virtual cell. But is it sufficient for this purpose? Does the data cover the major dimensions of SLC-relevant biological variation? Collectively, the four studies demonstrate how crucial recording the interplay between SLCs and all proteins or transcripts in the cell (Frommelt et al, 2025; Wiedmer et al, 2025) or recoding such an interplay in different metabolically relevant conditions are (Wolf et al, 2025). However, none of the here presented studies cover both dimensions—conditions and SLC-by-all—for a simple reason: experimental infeasibility. Perhaps lab-in-a-loop approaches that utilize the here reported data for computational predictions of ideal experiments that subsequently enable improved predictions can fill this data gap (Fig. 1). Even with such additional data pending, the SLC-focused omics layers reported in this issue of *Molecular Systems Biology* may spark attempts to generate what could become the representation of how solute carrier substrate transport is regulated across membranes of a virtual cell.
